# A Quantitative Curriculum Mapping of the Faculty of Pharmacy of Yeditepe University, Turkey: A Process to Assess the Consistency of a Curriculum with the Mission and Vision of an Institution and National Requirements

**DOI:** 10.3390/pharmacy7030078

**Published:** 2019-07-01

**Authors:** Filiz Esra Önen Bayram, Meriç Köksal

**Affiliations:** Department of Pharmaceutical Chemistry, Faculty of Pharmacy, Yeditepe University, Istanbul 34755, Turkey

**Keywords:** pharmacy education, curriculum, mapping, competency, program enhancement

## Abstract

The changing role of the pharmacist led to some improvements of pharmacy education worldwide these last years. Curricula have evolved and the content-based education has been converted into a competency-based education. The definition of a global practice-based competency framework by the International Pharmaceutical Federation (FIP) and the European Pharmacy Competencies Framework by the European the Quality Assurance in European Pharmacy Education and Training (PHAR-QA) project helps Universities to keep in with these changes. The National Council of Deans of Faculties of Pharmacy in Turkey also defined 169 competencies with their sub-competencies that have to be reached upon the completion of a pharmacy education program, yet it did not indicate how the faculties can measure if their curricula are consistent with these competencies. This study aims to provide a method for a quantitative mapping of a given curriculum in order to analyze if a curriculum fulfills the requirements defined by the National Deans Council. It also helps to easily determine the weaknesses and strengths of a program. Moreover, with this study, the consistency of the content of the courses with the mission and vision defined by an institution can be easily determined. Thus, this study can also be a useful tool for the revision and enhancement of a program according to institutional targets.

## 1. Introduction

Pharmacy education has to adapt to the changing role of the pharmacists whose responsibilities have been redefined according to the needs of the society, their practice being today much more patient-oriented [1,2]. A first adjustment has been carried out at the end of the 1990s via the Bologna process that aimed to harmonize education throughout Europe, converting existing programs into competency-based curricula, which consist of educational programs that define the abilities and skills that a student should acquire when graduating [3]. Thus, faculties of pharmacy of countries that adhered to the Bologna process adapted their programs and defined competencies delivered by their educational program. As no specific directives were given for the definition of the competencies, a need for a competency framework specific to pharmacy education has emerged [4]. This issue was addressed by the International Pharmaceutical Federation (FIP) who, in collaboration with the World Health Organization (WHO) and the United Nations Educational, Scientific and Cultural Organization (UNESCO), developed a global framework of practice-based competencies as a set of behavioral competencies defined around four main domains: pharmaceutical public health, pharmaceutical care, organization and management and professional/personal in 2012 [5].

Also in 2012, throughout the Pharmacy Education in Europe (PHARMINE) project, a survey conducted around European countries that gathered all the partners of pharmacy education (academic members along with students and practitioners from community pharmacies, hospital, and pharmaceutical industry) led to the elaboration of another framework of competencies [6]. This framework was further tuned during the Quality Assurance in European Pharmacy Education and Training (PHAR-QA) project to lead in 2016 to the European Pharmacy Competencies Framework that defines 50 competencies that have to be acquired by a freshly graduated European pharmacist [7,8,9,10,11].

In Turkey, the pharmacy education begins during the Ottoman Empire in 1839 with the foundation of the first pharmacy school by the Austrian pharmacist K. Ambros Bernard. In that time the education was in French and lasted, at the beginning, two, and a few years later, three years, application to the school requiring at least a six-month-long experience in a pharmacy. Almost 30 years later, in 1867, the first institution providing a pharmacy education in the Turkish language was founded. Certification from this institution was upon completion of a three-year-long education in addition to a three-year-long experience in a pharmacy. The foundation of this institution is followed by the creation of some other schools of pharmacy that have been further closed partially due to World War I. When the Turkish Republic is founded in 1923, the country has only one School of Pharmacy, which is related to the School of Medicine until 1933. It is a three-year-long education that is reformed in 1938 to be extended to four years. At the beginning of the 1960s a reform allowed the creation of independent pharmacy faculties and, in 1961, the Faculty of Pharmacy of Ankara University was founded. In 1962, the existing school of Pharmacy of Istanbul was also converted to the Faculty of Pharmacy of Istanbul University. Given an increasing need in the number of pharmacists throughout the country, the Turkish Ministry of Education allows to some private institutions to open private pharmacy schools that apply the curriculum of these two pharmacy faculties. A total of 8 private schools are created between 1964 and 1968, two of them being in Istanbul, two in Ankara, three in Izmir and one in Eskisehir. Some reforms expropriated these schools in 1971, 5 of which being transformed into Faculties [12,13,14,15,16]. Thus, from the beginning of the 1970s to the beginning of the 21st century there have been only seven faculties of pharmacy that were in charge of the education of pharmacists. 2000–2003 were years during which four new faculties were added to the existing seven, increasing their number to 11. The 11 faculties were subject to some national regulations that were redefined in 2008 where the competencies that a graduated pharmacist should acquire were stated, along with some compulsory subjects that had to be taught [17]. This regulation has been further modified in 2009 [18] and recently in 2018 [19]. According to the most recent regulation, the pharmacy diploma is delivered to students that has completed a ten-semester-long program that comprises at least four years of full time education (theoretical and practical) and a minimum of six-month-long internship that can be performed either in a pharmacy, hospital or a pharmaceutical or cosmetic company. Admission is possible via a national competitive exam and the faculties to which the students can register are determined according to the scores they have obtained.

Last decade constituted a turning point for pharmacy education in Turkey since more than 20 faculties have been founded throughout the whole country between 2010 and 2019. In terms of curricula faculties present some differences [20]. In order to ensure a harmonized education all around the country a study to determine a common core curriculum that defines the content of each compulsory course started in 2015. Additionally, Turkey has been a full member of the Bologna process since 2001 and as required in 2010, the Turkish National Qualification Framework has been defined consistent with those requirements. The Turkish Council of Higher Education that constituted the “Turkish Qualification Framework” demanded each University to define competencies to be acquired at the end of each curriculum and to associate them with the qualifications of the framework [21]. Thus, each faculty of pharmacy has defined its own set of competencies. To standardize the competencies throughout the country, the National Council of Deans of Faculties of Pharmacy has defined again in 2015, 169 competencies that have to be reached upon the completion of a pharmacy education program [22] and a further study has detailed these competencies into sub-competencies (an example of a competency with the related sub-competencies can be found in Table 2) [23]. These competencies define the technical skills that a freshly graduated pharmacist should acquire upon the completion of his/her education. The Council, however, did not indicate how faculties should measure the fulfillment of these outputs and there is an urgent need for a measurement tool to determine easily how much a curriculum matches the defined requirements and competencies. Thus, in this study, we aimed to develop a method that allows a quantitative mapping of a given curriculum through which its content and the targeted competencies can be analyzed and that can constitute a reference tool for the quantitative evaluation of a curriculum.

## 2. Materials and Methods

The study has been performed at the Faculty of Pharmacy of Yeditepe University using the curriculum applied during the 2017–2018 academic year with the contribution of the entire academic staff that is constituted of 34 full-time and six part-time faculty members.

### 2.1. Determination of Competency Scores

To determine the scores, firstly all the 63 compulsory courses of the academic program have been classified into 11 different groups (Table 1) [24]. Then a table, where all the competencies gathered with their sub-competencies constituted the lines, and the 11 groups constituted the columns was prepared using the Microsoft Office Excel 2010 software (a part of the table is given in Table 2).

The academic staff who was first asked to complete a written consent to participate to the study had then to relate each of its courses with the 169 competencies defined by the Council. For this task, the lecturer of each course evaluated with the other members of its department if their course content did meet or not the defined sub-competencies by scoring it with 1 if the sub-competency was met, or 0 if not. An average of all scores obtained for the sub-competencies gave then the score for the competency as a percentage, thus indicating quantitatively how much the competency is met by the group of interest (Table 3). Furthermore, the addition of the scores obtained for a competency by all the groups led to a total score that indicated how much the competency is met within the entire 2017–2018 academic program (last column of Table 3). It is important to notice that before the evaluation has been carried out, the table was first completed with the members of the department of pharmaceutical chemistry, as the authors are part of this department. Then, a training for scoring the competencies was provided to all faculty members and the partially completed table was also presented. Additionally, as the Faculty of Pharmacy of Yeditepe University is constituted of only 34 full-time and six part-time faculty members, the evaluation of the state of completion of competencies was performed during meetings that gathered all the faculty members of a given department (constituted of a maximum of five people) in the presence of the authors of the study that were also present in order to quickly answer to their questions if they had any while filling the table. Thus the scores were attributed as a common decision from all the members of the concerned departments.

### 2.2. Competency Heatmap

**Figure 1:**
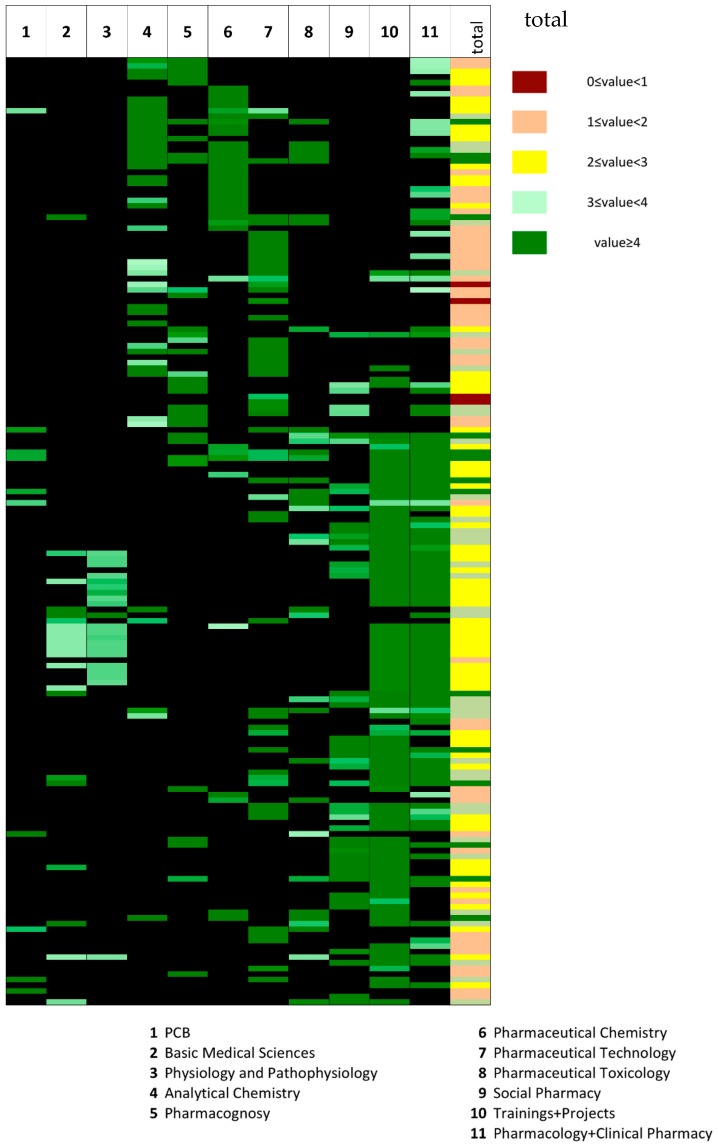
The competency heatmap. For values from column 1 to 11 a gradual scale of green has been applied from 0.01 to 1 and the cells with the value of 0 were colored in black to easily detect the competencies that have been evaluated as related to the courses of a given department. The last column titled as “total” was colored according to color grade given in the legend.

To visualize and analyze at a glance the state of fulfillment of each competency, the obtained scores were transferred into a heatmap. A scale of green color has been used to present the scores. The darkest green symbolizes a 100% fulfillment of a given competency that is gradually lightened when the percentages decrease. For a competency that was not related at all with a course group, that is to say that obtained the value of 0 for the competency, the box was colored in black. The final column of the map represents the total scores where the color scale varies from green to red where the darkest green represents the competencies with the highest total scores and the red regions refer to competencies that are not 100% met when the curriculum is considered as a whole. This column thereby helps to easily and quickly detect the strengths and weaknesses of the curriculum (Figure 1).

## 3. Results and Discussion

### 3.1. The State of Fulfillment of the Competencies

The measurement method was applied to the curriculum of the Faculty of Pharmacy of Yeditepe University (Istanbul, Turkey). This faculty has been founded in 2001 and at the time of the study is composed of 34 full-time and six part-time academic members and approximately 365 undergraduate students.

Its curriculum has first been constituted in 2001 where topics indicated by the Turkish Council of Higher Education were included. The initial curriculum has been revised in 2005, 2012, and 2016 according to the needs that were identified by the faculty members and the changes in regulations. Today’s curriculum of the Faculty of Pharmacy of Yeditepe University is divided into ten semesters and comprises 63 compulsory courses (theoretical, practical courses, and internships) and six elective courses that the student should take among 48 possibilities. The student should also complete three free elective courses that can be taken from any faculty of the University according to his career goals or personal interests. Every student should also take five courses that deal with global and Turkish History and Turkish Literature in order to graduate. Thus, graduation from this faculty is possible with the completion of 300 ECTS (European Credit Transfer and Accumulation System). This study has been carried out on the 63 compulsory courses since the content of these courses is expected to be similar to the ones taught at other faculties. As this study constitutes a first step for the evaluation of the curriculum, it has been carried out only with the contribution of academic members. A more exhaustive study that will take into account the evaluations performed by students and graduates will be accomplished later.

The state of fulfillment of the competencies defined by the Council can be determined by analyzing the last column of Figure 1 along with Table 4 that gives the distribution of competencies according to their total scores. When the data are observed, it is possible to see that none of the competencies presents a total score of 0, indicating that this program’s core courses satisfy all the requirements defined by the Council. Also, the results show that while four of the 169 competencies were not found to be 100% fulfilled (dark red in total column, Figure 1), 49 were recorded to show a score between 1 and 2 (orange), 65 a score comprised between 2 and 3 (yellow), 36 a score within the 3–4 range (light green), and 15 that were greater than 4 (dark green) (Figure 1 and Table 4).

This analysis allowed an easy identification of the strengths and weaknesses of the program. Thus, the four competencies for which the score was less than 1 indicated the weakest parts to be strengthen and the 15 competencies with scores greater than 4 indicated the strengths and/or the redundancies of this curriculum.

The four competencies for which the score did not reach the value of 1 were two that dealt with biotechnology (89% for the 41st competency: prepares biotechnological products, 92% for the 44th competency: develops a drug formulation) and two that concerned microbiological experimentation (50% for the 61st competency: performs microbiological controls during drug production processes, 93% for the 62nd competency: performs the microbiological analysis of cosmetics) (Table 5).

As the results indicated a weakness in the field of microbiology, the Faculty Education Committee decided to reorganize the content of its curriculum in order to be able to provide to its students the required know-how. Thus, the existing PHAR 427 Cosmetology + Lab course has been reviewed and its syllabus redefined to incorporate some new topics that focus on microbiological analyses. The other weakness that has been determined was in the department of pharmaceutical technology of the faculty. The finding that the drug formulation competency (competency #44) was not 100% fulfilled was an unexpected result that forced the department’s academic members to revise the content of their courses. Additionally, to strengthen this department that constituted three assistant professors at the time of the study, a full-time professor position was created and an academician was hired three months after the weakness was detected. The revised curricula were applied starting from the 2018–2019 academic year.

The 15 competencies that constituted the strength of the program and for which the total score was greater than 4 are gathered in Table 6. These results first indicated that most of the competencies were related to topics that are thought in clinical pharmacy courses (68, 71, 72, 76, 78, 114, 124, 130, 147). The clinical pharmacy courses of the Faculty of Pharmacy of Yeditepe University are courses during which students are carrying out some case studies with the possibility of directly visiting patients in hospitals. This practice is not common in pharmacy education in Turkey and constitutes one of the specificities of Yeditepe University since 2005. High scores for these competencies thus pointed that the content of the curriculum of the faculty was consistent with its mission and vision (http://eczacilik.yeditepe.edu.tr/en/vision-mission). Another group of competencies for which the scores were very high were related to analytical techniques (12, 18, 19, 154). The reason of such a finding can be the existence of some redundancies in the curriculum, a possibility that has been discussed with Faculty Education Committee that decided to determine the chapters that were related to these competencies for each department in order to revise the content of the courses if redundancies are detected.

### 3.2. The Specificities of the Curriculum of the Faculty of Pharmacy of Yeditepe University

One of the specificities of the curriculum of the Faculty of Pharmacy of Yeditepe University is that, in addition to providing an excellent-level pharmacy education, it has a mission of preparing leader pharmacists for the Turkish pharmaceutical industry [25]. However, when the scores of the competencies related to this mission were analyzed, they were found to be generally comprised between 2 and 3. Actually, the analysis that was carried out comprised only the compulsory courses of the curriculum and thus did not display the impact of the elective courses provided to fifth-year students that aim to develop their entrepreneurial competencies. That could explain why this analysis does not show the emphasis put on the pharmaceutical industry by this program. Thus our results pointed out the importance of the analysis of the elective courses to be able to highlight the competencies that are specifically developed in a program.

Furthermore, to increase the scores of industry-related competencies, the faculty administration decided to enlarge its collaborative works with the pharmaceutical industry and to revise its curriculum content in order to:

- increase the competent workforce through the employment of pharmacists in the pharmaceutical industry;
- provide leading pharmacists who can hold senior positions in all units of national and international pharmaceutical companies; and
- strengthen the collaboration with the pharmaceutical industry, as indicated in the vision of this Faculty.

### 3.3. Comparing Curricula of Different Institutions

This methodology can constitute a valuable tool for the evaluation of the curricula of different institutions, as scoring a competency through sub-competencies can constitute a precise and tunable tool. Indeed, when evaluating the status of fulfillment of a given competency, the details of what is aimed to be achieved can be defined with a number of sub-competencies by the author of the survey. The definition of the sub-competency would be designed so that the evaluator can with no ambiguity answer with a “yes” that is scored with 1 or “no” scored with 0. Thus, the average obtained for a competency results in a percentage that indicates how much the competency is met. This process should avoid the differences in the interpretation of the values of a Likert-type scale as suggested in the literature [26,27,28]. Indeed, the problem of differences in scorings during competency evaluation was identified by Gmeiner et al. when the authors carried out a curriculum mapping study using the PHAR-QA framework even when a five-point Likert-type scale was used (0 = not covered, 1 = poor, 2 = fair, 3 = good, 4 = very good) [29]. Detailing competencies into sub-competencies that will be evaluated as being covered or not, and thus scored as 1 or 0 would minimize bias allowing a fairer comparison of curricula of different institutions. The definition of detailed sub-competencies and their binary evaluation would also help to quickly and easily determine the gaps that have to be filled in order to meet the minimum requirements of a given competency framework.

## 4. Conclusions

This study demonstrated that the curriculum of the Faculty of Pharmacy of Yeditepe University fulfills the requirements defined by the National Dean Council. It also helped to easily determine the weaknesses and strengths of the program, using a quantitative measurement. The scores that were calculated allowed not only to point out the competencies that are provided with a satisfactory level, but also the ones that have to be strengthened. Moreover, through this investigation, the consistency of the content of the courses with the mission and vision defined by an institution can be easily determined and, thus, such an analysis can help to determine how a program can be revised and enhanced according to institutional targets.

## Figures and Tables

**Table 1:** Classification of the courses of the academic program—Yeditepe University Faculty of Pharmacy Course Distribution.

Course Code	Course Name	Group
PHAR 105	General Chemistry	Physics-Chemistry-Biology (PCB)
PHAR 103	Molecular Genetics and Biology	PCB
MATH 160	Introduction to Calculus	PCB
MDP 102	Biostatistics	PCB
PHAR 121	Physics for Health Sciences	PCB
MDP 220	Pharmaceutical Microbiology	Basic Medical Sciences
MDP 221	Biochemistry	Basic Medical Sciences
PHAR 431	Clinical Biochemistry	Basic Medical Sciences
MDP 120	Anatomy	Basic Medical Sciences
MDP 240	Immunology	Basic Medical Sciences
PHAR 156	Basic First Aid	Basic Medical Sciences
PHAR 126	Public Health	Basic Medical Sciences
PSY 220	Psychology for Health Sciences	Basic Medical Sciences
PHAR 142	Physiology	Physiology & Pathophysiology
PHAR 233	Pathophysiology	Physiology & Pathophysiology
PHAR 255	Pharmaceutical Analytical Chemistry 1	Analytical Chemistry
PHAR 256	Pharmaceutical Analytical Chemistry 2 + Lab.	Analytical Chemistry
PHAR 496	Drug Analysis	Analytical Chemistry
PHAR 234	Medicinal Plants + Lab	Pharmacognosy
PHAR 321	Pharmacognosy 1 + Lab. 1	Pharmacognosy
PHAR 322	Pharmacognosy 2 + Lab. 2	Pharmacognosy
PHAR 425	Phytotherapy and Applications	Pharmacognosy
PHAR 114	Pharmaceutical Organic Chemistry 1	Pharmaceutical Chemistry
PHAR 213	Pharmaceutical Organic Chemistry 2 + Lab.	Pharmaceutical Chemistry
PHAR 212	Pharmaceutical Chemistry 1 + Lab. 1	Pharmaceutical Chemistry
PHAR 311	Pharmaceutical Chemistry 2 + Lab. 2	Pharmaceutical Chemistry
PHAR 318	Pharmaceutical Chemistry 3	Pharmaceutical Chemistry
PHAR 303	Pharmaceutical Technology 1 + Lab. 1	Technology
PHAR 308	Pharmaceutical Technology 2 + Lab. 2	Technology
PHAR 427	Cosmetology + Lab.	Technology
PHAR 412	Pharmacokinetics	Technology
PHAR 414	Biopharmaceutics	Technology
PHAR 422	Pharmaceutical Biotechnology	Technology
PHAR 494	Drug Quality Management	Technology
PHAR 526	New Drug Delivery Systems	Technology
PHAR 154	Nutrition and Health	Toxicology
PHAR 312	Pharmaceutical Toxicology + Lab.	Toxicology
PHAR 413	Clinical Toxicology	Toxicology
PHAR 433	Preventive Health	Toxicology
PHAR 118	Communication Skills	Social Pharmacy
PHAR 415	Pharmacy Management	Social Pharmacy
PHAR 436	Pharmaceutical History and Deontology	Social Pharmacy
PHAR 521	Pharmaceutical Regulations	Social Pharmacy
PHAR 522	Pharmacoeconomics and Pharmacoepidemiology	Social Pharmacy
PHAR 101	Introduction to Pharmacy	Trainings + Projects
PHAR 124	Computer Usage in Pharmacy	Trainings + Projects
PHAR 408	Literature and Projects	Trainings + Projects
PHAR 293	Practical Pharmacy Training 1	Trainings + Projects
PHAR 294	Practical Pharmacy Training 2	Trainings + Projects
PHAR 295	Community Pharmacy Training 1	Trainings + Projects
PHAR 391	Community Pharmacy Training 2	Trainings + Projects
PHAR 491	Optional Training	Trainings + Projects
PHAR 501	Graduation Project 1	Trainings + Projects
PHAR 502	Graduation Project 2	Trainings + Projects
PHAR 510	Graduation Training	Trainings + Projects
PHAR 232	Pharmacology 1	Pharmacology + Clinical Pharmacy
PHAR 331	Pharmacology 2	Pharmacology + Clinical Pharmacy
PHAR 316	Pharmacology 3	Pharmacology + Clinical Pharmacy
PHAR 401	Clinical Pharmacy Applications 1	Pharmacology + Clinical Pharmacy
PHAR 402	Clinical Pharmacy Applications 2	Pharmacology + Clinical Pharmacy
PHAR 404	Clinical Pharmacy and Pharmaceutical Care 2	Pharmacology + Clinical Pharmacy
PHAR 403	Clinical Pharmacy and Pharmaceutical Care 1	Pharmacology + Clinical Pharmacy
PHAR 416	Pharmaccotherapy	Pharmacology + Clinical Pharmacy

**Table 2:** Table used to determine the fulfillment status of competency #1.

Competency #	Competency	Sub-Competencies	How to Reach?	PCB	Basic Medical Sciences	Physiology and Pathophysiology	Analytical Chemistry	Pharmacognosy	Pharmaceutical Chemistry	Pharmaceutical Technology	Pharmaceutical Toxicology	Social Pharmacy	Trainings + Projects	Pharmacology + Clinical Pharmacy
1	Can obtain an active substance from natural sources.	Determines the suitable natural source.	Differentiated natural sources.	0	0	0	0	1	0	0	0	0	0	1
1			Identifies the natural source.	0	0	0	0	1	0	0	0	0	0	0
1			Obtains the natural source.	0	0	0	0	1	0	0	0	0	0	0
1		Designs the separation technique.	Determines the suited extraction method.	0	0	0	1	1	0	0	0	0	0	0
1			Defined the operation steps of an extraction.	0	0	0	1	1	0	0	0	0	0	0
1			Prepares the extraction conditions.	0	0	0	1	1	0	0	0	0	0	0
1			Determines the separation (chromatographic) method.	0	0	0	1	1	0	0	0	0	0	0
1			Defines the operation steps of the separation process	0	0	0	1	1	0	0	0	0	0	0
1			Provides the separation conditions.	0	0	0	1	1	0	0	0	0	0	0
1		Carries out the separation technique.	Use the instrument suited to the determined method.	0	0	0	1	1	0	0	0	0	0	0
1			Uses the solvent system suited to the determined method.	0	0	0	1	1	0	0	0	0	0	0
1			Uses the adsorbant suited to the determined method.	0	0	0	1	1	0	0	0	0	0	0
1			Uses the isolation method suited to the determined method.	0	0	0	1	1	0	0	0	0	0	0
1			Uses the purification method suited to the determined method.	0	0	0	1	1	0	0	0	0	0	0
1		Analyses the active substance.	Determines the structure analysis methods for the purified substances.	0	0	0	1	1	0	0	0	0	0	0
1			Performs the structural analysis.	0	0	0	1	1	0	0	0	0	0	0
1			Determines the physocal properties of the active substance.	0	0	0	1	1	0	0	0	0	0	0
1			Explains their suitability to standarts.	0	0	0	1	1	0	0	0	0	0	0
1		Reports the performed technique and its results.	Evaluated data.	0	0	0	1	1	0	0	0	0	0	1
1			Reports about the performed method.	0	0	0	1	1	0	0	0	0	0	0
1			Reports the results.	0	0	0	1	1	0	0	0	0	0	0
**1 Average**			**Total score**	0	0	0	0.857	1	0	0	0	0	0	0.095

The first eleven columns indicate the average score of for each competency obtained as the average of scores that were attributed for each sub-competency (cf. last row of Table 2). The last “row total” column gives the sum of the average scores obtained by all the eleven groups.

**Table 3:** Average scores of competencies obtained by each department and the total score of a competency.

Competency #	PCB	Basic Medical Sciences	Physiology and Pathophysiology	Analytical Chemistry	Pharmacognosy	Pharmaceutical Chemistry	Pharmaceutical Technology	Pharmaceutical Toxicology	Social Pharmacy	Training + Projects	Pharmacology + Clinical Pharmacy	Row Total
1 Average	0	0	0	0.85714286	1	0	0	0	0	0	0.0952381	1.95238095
2 Average	0	0	0	0.6	1	0	0	0	0	0	0.1	1.7
3 Average	0	0	0	1	1	0	0	0	0	0	0.14285714	2.14285714
4 Average	0	0	0	1	1	0	0	0	0	0	0	2
5 Average	0	0	0	0	1	0	0	0	0	0	1	2
6 Average	0	0	0	0	0	1	0	0	0	0	0	1
7 Average	0	0	0	0	0	1	0	0	0	0	0.16666667	1.16666667
8 Average	0	0	0	1	0	1	0	0	0	0	0	2
9 Average	0	0	0	1	0	1	0	0	0	0	0	2
10 Average	0.25	0	0	1	0	0.75	0.25	0	0	0	0	2.25
11 Average	0	0	0	1	0	1	1	0	0	0	0	3
12 Average	0	0	0	1	1	0.9	0	1	0	0	0.2	4.1
13 Average	0	0	0	1	0	1	0	0	0	0	0.2	2.2
14 Average	0	0	0	1	0	1	0	0	0	0	0.22222222	2.22222222
15 Average	0	0	0	1	1	0	0	0	0	0	0	2
16 Average	0	0	0	1	0	1	0	1	0	0	0	3
17 Average	0	0	0	1	0	1	0	1	0	0	0.75	3.75
18 Average	0	0	0	1	1	1	0	1	0	0	1	5
19 Average	0	0	0	1	1	1	1	1	0	0	0	5
20 Average	0	0	0	1	0	1	0	0	0	0	0	2
21 Average	0	0	0	0	0	1	0	0	0	0	0	1
22 Average	0	0	0	1	0	1	0	0	0	0	0	2
23 Average	0	0	0	1	0	1	0	0	0	0	0	2
24 Average	0	0	0	0	0	1	0	0	0	0	0.5	1.5
25 Average	0	0	0	0	0	1	0	0	0	0	0.33333333	1.33333333
26 Average	0	0	0	0.4	0	1	0	0	0	0	0	1.4
27 Average	0	0	0	1	0	1	0	0	0	0	0	2
28 Average	0	0	0	0	0	1	0	0	0	0	0.75	1.75

0 ≤ score < 1 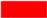         1 ≤ score < 2 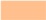         2 ≤ score < 3 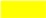         3 ≤ score < 4 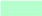         score ≥ 4 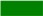

**Table 4:** Score distribution of competencies.

Competency Score	Number of Competencies	Competency #
0 ≤ score < 1	4	41, 44, 61, 62
1 ≤ score < 2	49	1, 2, 6, 7, 21, 24-26, 28, 31-38, 40, 42, 43, 45-48, 51, 52, 54, 55, 65, 66, 80, 108, 119, 120, 131-133, 139, 142, 149, 151, 157-160, 163, 164, 167, 168
2 ≤ score < 3	65	3-5, 8-10, 12-15, 20, 22, 23, 27, 49, 57, 59, 60, 67, 70, 73-75, 77, 81, 82, 84, 88-90, 92, 94-98, 101-107, 109-113, 121-123, 125, 127, 136-138, 144-146, 148, 150, 152, 156, 161, 166
3 ≤ score < 4	36	11, 16, 17, 30, 39, 50, 53, 56, 63, 64, 69, 79, 83, 85-87, 91, 93, 99, 100, 115-118, 126, 128, 129, 134, 135, 140, 143, 153, 155, 162, 165, 169
4 ≤ score < 5	11	12, 29, 68, 76, 78, 114, 124, 130, 141, 147, 154
score ≥ 5	4	18, 19, 71,72

**Table 5:** Competencies that are not 100% fulfilled.

Competency #	Competency	Score
41	Prepares biotechnological products	89%
44	Develops a drug formulation.	92%
61	Performs microbiological controls during drug production processes	50%
62	Performs the microbiological analysis of cosmetics.	93%

**Table 6:** Competencies with scores greater than 4.

Competency #	Competency	Score
18	Uses quantitative techniques for drug analysis.	5
19	Uses the UV spectrophotometric techniques for drug analysis.	5
71	Determines and evaluates drug-drug interactions.	5
72	Determines and evaluates drug-nutrient interactions.	5.7
12	Performs the thin layer chromatography technique (TLC) in drug analysis.	4.1
29	Defines drug metabolism.	4.75
68	Provides drug information.	4.2
76	Controls drug doses.	4
78	Determines and evaluates drugs’ adverse effects.	4.3
114	Helps patients to consult and take benefit from the existing health care services.	4
124	Submits and manages drugs subjected to controlled prescription (red and green coloured prescriptions).	4
130	Provides information concerning the rational and safe use of a blood glucose meter.	4.1
141	Performs literature search and sourcing.	4
147	Raises awareness concerning drugs and health.	4.3
154	Performs chromotographic techniques to define active substances and their metabolites.	4
